# Practical Hints for Dentists

**Published:** 1883-03

**Authors:** J. A. Robinson

**Affiliations:** Jackson, Mich.


Article IV.

### PRACTICAL HINTS FOR DENTISTS.

BY J. A. ROBINSON, D.D.S., JACKSON, MICH.

The greatest Teacher the world has ever known said on one occasion to a sinner, "Go thy way and sin no more." There is no evidence that the command was obeyed, but the accusers of the woman went out until there was none left to accuse. The probability is that all the parties were benefited by the saying that was spoken by the man who spake as never man spoke. The three greatest sinners in dentistry to be treated by the method laid down by the great Teacher, are tooth extraction, amalgam fillings, and artificial dentures on rubber. It is very evident that there will always be sinners in the world, and the great thing to learn is, what it is best to do with them. The sinners of the world are really more useful than some of us at first thought are willing to admit. The veriest vagabond may be an expert swimmer and save a child from drowning. It sometimes happens that the most worthless wretch in all the community saves the lives of men and women from a great conflagration. If we can utilize an evil it is no longer an evil. Strictly speaking there is no positive evil in the world, but as the excess of fire is a conflagration, the excess of the soft wind a tornado, the excess of blood in any portion of the body a congestion, plethory and death, so the very ills of life stimulate us to exertion to overcome those ills, as the abuse of amalgum stimulates us to the endeavor to find a substitute that is less injurious. The sting of a toothache leads to an attempt to cure the pain, and the excruciating torture of inflamed tissues, pent up within the hard structure of a tooth, being, as Burns has said, "The hell of all diseases," has made us what we are as a profession, and is stimulating us to try to convince the world that it is better to endure the ills we have than fly to others that we know not of.

The world must be taught that there can be no artificial substitute which can completely fill the place of that which nature has provided for us.

To save is to preserve from injury of any kind; so we cannot save any person or thing without doing that person or thing good. The highest function of the dentist is, therefore, to preserve the natural organs, and, when that is impossible, to utilize the imperfect things we have, and to substitute more perfect things as time and experience shall lead us up to higher excellence and attainment.

All amalgamated metals are so far failures, but they may be made less so by manipulation and by neutralizing the evil properties as far as possible. That mercury is necessary to form an amalgam with silver is patent to every practitioner. That mercury will not amalgamate with platinum is, I believe, a conceded fact; hence all amalgams having a combination of platinum are in no way benefitted by it. The platinum occupies space that could be better filled by some metal that would chemically unite to form a more homogeneous mass.

Tin foil is recommended and frequently used to take up the superabundance of mercury, but the use of a plugger of pure tin to pack the amalgam is better, inasmuch as you can begin to take away the mercury from the amalgam when the cavity is but partially filled.

After using, the point of the tin plugger can be heated and the mercury partially sublimated. When the end of the instrument is so thoroughly amalgamated as to be useless, it may be broken off and reshaped with the hammer, and thus made fit for a new career of usefulness. I have some made of equal parts of tin and silver that answer a good purpose. They are more rigid than tin alone, and in that particular better. Imbed a large size plugger in moulding sand and cast one for yourself and try it.

All rubber plates, from some cause not yet determined, produce an inflamed condition of the mucus surface of the mouth, and any invention or discovery that will preclude that condition will be a lasting benefit to the profession, for rubber has too many good qualities to be abandoned if it be possible to use it without positive injury. The method of making such plates is disagreeable and dirty, and the task is at best an irksome one.

A convenient way to warm rubber for packing is to heat it on the chimney of a kerosene lamp. It will stick to the chimney, while the heat can be easily regulated so that the danger of injury to the rubber from overheating is readily avoided.

Provide a box, into which place a mixture of one pound of soda bicarbonate for every two quarts of pine sawdust. When a flask is removed from the vulcanizer place it in this, and with a dampened shoe brush clean it before opening. Any stains upon the hands may be removed by washing them in the same mixture. A piece of a newspaper makes a very convenient holder when handling the flasks.

If the vulcanizer leaks steam, cover the packing with a thin coat of stove-blacking made into a paste with water. This will last for years if the vulcanizer be in constant use.

Anything prematurely done is not well done. The extraction of comparatively sound teeth under the pretense that they would not last long at any rate, and must be eventually lost, is a crime against humanity.

---

Iodoform for Ascaris Lumbricoides.—The St. Petersburg *Med. Woch.*, December 30, 1882, says that Dr. Schildowsky has employed iodoform successfully in three cases of ascaris, and recommends that a further trial should be given of the remedy. He gives to an adult one grain with ten grains of bicarbonate of soda three times a day, and a quarter of a grain to a child.

